# What is changing in the adjuvant treatment of melanoma?

**DOI:** 10.18632/oncotarget.22988

**Published:** 2017-12-06

**Authors:** Paolo A. Ascierto, Giuseppe Palmieri, Helen Gogas

**Affiliations:** Paolo A. Ascierto: Istituto Nazionale Tumori IRCCS Fondazione “G. Pascale”, Napoli, Italy

**Keywords:** melanoma, adjuvant therapy, nivolumab, ipilimumab, BRAF inhibitors

Until recently, interferon (IFN)-α was the only drug approved as adjuvant therapy for patients with melanoma at high-risk of recurrence after surgical resection. A recent meta-analysis provided further evidence that adjuvant IFN-α is associated with a significant reduction in the risk of relapse and improved survival [1]. Moreover, there was no evidence that the benefits of IFN-α were dependent on dose or duration of treatment or disease stage, although patients without ulcerated tumours did not appear to benefit. However, the use of IFN-α as adjuvant therapy in melanoma remains contentious, with questions over patient selection and optimal treatment regimen as well as concerns over toxicity. As such, there remains a need for treatment options that are more effective and better tolerated.

The advent of checkpoint inhibitors has revolutionized the treatment of advanced melanoma and their use as adjuvant therapy is now a focus of attention. In a phase III trial of 951 patients who had undergone complete resection of stage III cutaneous melanoma, the anti-cytotoxic T-lymphocyte-associated antigen (CTLA)-4 antibody ipilimumab, at a dose of 10 mg/kg, resulted in a significantly higher rate of 5-year overall survival (OS) than placebo, with a 28% risk reduction for death (hazard ratio [HR], 0.72; 95.1% confidence interval [CI]: 0.58-0.88; *p* = 0.001) [2]. However, toxicity was a major concern with almost half of the patients receiving ipilimumab (48%) discontinuing treatment because of drug-related toxicity. Despite this, ipilimumab was approved in the US for adjuvant treatment of melanoma in 2015.

More data on the potential adjuvant use of checkpoint inhibitors, as well as that of targeted agents, were presented at the ESMO 2017 congress. In the phase III CheckMate 238 trial, ipilimumab 10 mg/kg was compared with the anti-programmed death (PD)-1 antibody nivolumab (3 mg/kg) in 906 patients after complete resection of stage IIIB-IV melanoma [3]. Recurrence-free survival (RFS) at one year was 70.5% (95% CI: 66.1-74.5) in the nivolumab group compared with 60.8% (95% CI: 56.0-65.2) in the ipilimumab group (HR for disease recurrence or death, 0.65; 97.56% CI: 0.51-0.83; *p* < 0.001). The RFS benefit for nivolumab over ipilimumab was observed in several subgroups, including those categorized by PD-L1 expression, BRAF status or disease stage. Nivolumab was also better tolerated, with fewer treatment-related grade 3-4 adverse events (14.4% *vs* 45.9% of patients) and treatment-related discontinuations (9.7% *vs* 42.6%) compared with ipilimumab.

In the BRIM8 trial, one year of adjuvant monotherapy with the BRAF inhibitor vemurafenib provided a significant improvement in disease-free survival (DFS) (46% risk reduction, *p* = 0.0010) compared with placebo in 314 patients with resected stage IIC, IIIA or IIIB BRAF-mutated melanoma [4]. Vemurafenib also resulted in a significant reduction in distant metastasis-free survival (DMFS) in these patients. However, in a separate cohort of 184 patients with stage IIIC BRAF-mutated melanoma, the increase in median DFS with adjuvant vemurafenib was not statistically significant (23.1 *vs* 15.4 months; HR 0.80, 95% CI: 0.54-1.18; *p* = 0.2598). Treatment was generally well tolerated, and there wasn’t an increase in secondary skin cancers (cutaneous squamous cell carcinoma and keratoacanthoma) that are known to be associated with vemurafenib.

BRAF inhibition was also assessed in combination with MEK inhibition. In the Combi-AD trial of adjuvant dabrafenib plus trametinib in stage III BRAF-mutated melanoma, estimated 3-year RFS was 58% in 438 patients randomized to combination therapy versus 39% in 432 patients receiving placebo (HR for relapse or death, 0.47; 95% CI: 0.39-0.58; *p* < 0.001) [5]. Three-year OS was 86% in the combination group versus 77% in the placebo group (HR for death, 0.57; 95% CI: 0.42-0.79; *p* = 0.0006). However, 41% of patients had grade 3-4 adverse events and 26% discontinued treatment because of toxicity related to combination therapy.

Other studies reported at ESMO suggested a potential benefit of neoadjuvant therapy with dabrafenib plus trametinib [6] or combined ipilimumab plus nivolumab [7]. Interestingly, data presented at ASCO 2017 from a study of neoadjuvant dabrafenib plus trametinib suggested that patients with an upregulated baseline immuno-related gene signature were more likely to achieve a response while patients who progressed had an increase of PD-1 and TIM-3 in the infiltrating lymphocytes [8]. As such, the benefit of targeted therapy may also be explained by an immunological mechanism of action.

Based on these new results, IFN-α can no longer be considered the optimal adjuvant treatment choice in melanoma. Both nivolumab and combined dabrafenib plus trametinib offer increased potential to prevent metastatic disease after resection. Nivolumab has been shown to be more effective and less toxic than ipilimumab while dabrafenib plus trametinib appears to be superior to BRAF inhibitor monotherapy with vemurafenib, although vemurafenib may have a role in stage IIC disease. Both immunotherapy and targeted therapy were shown to be active in patients with minimal residual disease, and the choice to use one or the other will probably be made on the basis of patient characteristics (stage, BRAF status, safety profile, patient convenience). However, in the next year, the new AJCC system which includes different definitions among stage III disease, will become operative, For this reason, such data should also be put in the context of this new classification.

Other ongoing studies, in particular the US Intergroup E1609 trial of high-dose interferon versus ipilimumab at 10 mg/kg or 3 mg/kg, the KEYNOTE 054 trial of pembrolizumab versus placebo, the SWOG 1404 trial of pembrolizumab versus high-dose interferon, and the CheckMate 915 trial of combined ipilimumab plus nivolumab versus nivolumab alone, should provide further data on the role of adjuvant immune- and targeted therapy.
